# Protection against renal ischemia–reperfusion injury *in vivo* by the mitochondria targeted antioxidant MitoQ

**DOI:** 10.1016/j.redox.2015.04.008

**Published:** 2015-04-29

**Authors:** Anna J. Dare, Eleanor A. Bolton, Gavin J. Pettigrew, J. Andrew Bradley, Kourosh Saeb-Parsy, Michael P. Murphy

**Affiliations:** a Medical Research Council Mitochondrial Biology Unit, Cambridge BioMedical Campus, Hills Road, Cambridge CB2 0XY, UK; b Department of Surgery, University of Cambridge, NIHR Cambridge Biomedical Research Centre, Cambridge CB2 0QQ, UK

**Keywords:** Kidney, Ischemia–reperfusion injury, Mitochondria, Oxidative damage, Mitochondria targeted antioxidants, MitoQ

## Abstract

Ischemia–reperfusion (IR) injury to the kidney occurs in a range of clinically important scenarios including hypotension, sepsis and in surgical procedures such as cardiac bypass surgery and kidney transplantation, leading to acute kidney injury (AKI). Mitochondrial oxidative damage is a significant contributor to the early phases of IR injury and may initiate a damaging inflammatory response. Here we assessed whether the mitochondria targeted antioxidant MitoQ could decrease oxidative damage during IR injury and thereby protect kidney function. To do this we exposed kidneys in mice to *in vivo* ischemia by bilaterally occluding the renal vessels followed by reperfusion for up to 24 h. This caused renal dysfunction, measured by decreased creatinine clearance, and increased markers of oxidative damage. Administering MitoQ to the mice intravenously 15 min prior to ischemia protected the kidney from damage and dysfunction. These data indicate that mitochondrial oxidative damage contributes to kidney IR injury and that mitochondria targeted antioxidants such as MitoQ are potential therapies for renal dysfunction due to IR injury.

## Introduction

The kidney is vulnerable to ischemia–reperfusion (IR) injury during a number of clinically important scenarios, including hypotension, sepsis, surgical procedures such as partial nephrectomy and cardiac bypass surgery, as well as during kidney transplantation [1,2]. The clinical consequences of renal IR injury – or ischemic acute kidney injury (AKI) as the clinical syndrome is called – depend on the severity of the injury, and range from minor changes in renal function to a requirement for dialysis or transplantation [3]. Ischemic AKI is a major cause of morbidity and mortality [4], for example it can predispose to chronic kidney disease and contributes to delayed graft function and rejection following transplantation [5]. Currently there is no effective pharmacological strategy to address the underlying pathophysiology of ischemic AKI.

As ischemic AKI often occurs in high-risk patient populations as a result of predictable interventions such as cardiac bypass surgery or following kidney transplantation, pre-treatments that protect the kidney from IR injury are feasible. While protective agents such as N-acetyl cysteine have shown some efficacy against AKI caused by radiological contrast agents [6], progress towards a general strategy to decrease ischemic AKI has been frustratingly slow. To address this unmet clinical need we focused on pharmacological strategies aimed at protecting mitochondria, as damage to these organelles is central to IR injury [7]. During renal ischemia, oxygen deprivation depletes intracellular ATP, inactivates oxidative phosphorylation and leads to a compensatory switch to anaerobic metabolism [8]. Re-introduction of oxygen at reperfusion dramatically increases the generation of the damaging reactive oxygen species superoxide and hydrogen peroxide from mitochondria [9–11] damaging cellular lipids, proteins and DNA [9,12]. This disrupts mitochondrial ATP supply and in conjunction with calcium uptake can induce the mitochondrial permeability transition pore and thus cell death [13,14]. In addition, mitochondrial damage can activate inflammasome formation, augmenting the innate inflammatory response to tissue damage in the hours following the initial insult [15–18]. Therefore mitochondrial oxidative damage is a major factor in kidney IR injury.

**Fig. 1:**
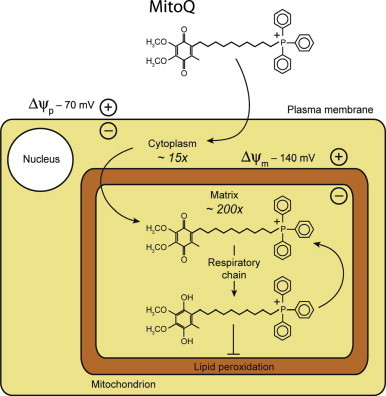
Accumulation of MitoQ within kidney mitochondria driven by the membrane potential. MitoQ accumulates within the cell driven by the plasma membrane potential (∆*Ψ_p_*), then further accumulates within mitochondria driven by mitochondrial membrane potential (∆*Ψ_m_*). In the mitochondrial matrix, MitoQ is reduced to the active antioxidant form, ubiquinol, by the respiratory chain preventing oxidative damage such as lipid peroxidation. Antioxidant activity generates the ubiquinone form, which is then recycled back to ubiquinol by the respiratory chain.

A pharmacological method to decrease mitochondrial oxidative damage is thus an appealing prospect for kidney IR injury [19]. Conventional antioxidants have not been effective clinically, in large part because they are not taken up by the mitochondria *in vivo* [20,21]. To overcome this limitation we developed mitochondria targeted antioxidants, the prototype of which is MitoQ [22]. This molecule comprises a lipophilic triphenylphosphonium (TPP) cation covalently linked by an aliphatic 10-carbon chain to an antioxidant ubiquinone moiety [23]. The TPP lipophilic cation passes rapidly through biological membranes and its positive charge drives the extensive accumulation of these molecules into mitochondria, due to the large mitochondrial membrane potential [22]. The ubiquinone moiety is activated and recycled by complex II within mitochondria and acts as a chain breaking antioxidant to prevent oxidative damage [20,23,24]. This combination of extensive uptake into mitochondria [23], recycling of the antioxidant moiety [25] and its location on the matrix facing surface of the mitochondrial inner membrane [26] makes MitoQ many thousand fold more potent at preventing oxidative damage than untargeted antioxidants [23] (Fig. 1). MitoQ has been used extensively in animal models of human pathologies [22] where it has been shown to be effective following oral, intravenous or intraperitoneal delivery [22]. In addition, MitoQ has been used in two Phase II trials in humans and been shown to be safe and well tolerated long term [27] and effective at preventing liver inflammatory damage in a small study of hepatitis C virus-patients [28].

The use of MitoQ for AKI is supported by the fact that MitoQ is rapidly cleared from the plasma following intravenous administration and accumulates within the kidney [29,30]. Furthermore, MitoQ decreases IR injury in other organs [15,31,32] and protects the kidney against damage during cold storage [33] and from diabetic nephropathy [34]. Together these findings suggest that MitoQ may be an effective therapy for the acute kidney IR injury. Here we have determined if the mitochondrial damage and kidney dysfunction caused by IR injury could be decreased by MitoQ. To do this we used a mouse model of bilateral renal ischemia, followed by up to 24 h reperfusion. We found that IV administration of MitoQ 15 min prior to ischemia was protective against kidney IR injury.

## Materials and methods

### *In vivo* mouse model of renal ischemia–reperfusion injury

Male C57BL/6 mice (25±10 g) from Charles River Laboratories were maintained in specific pathogen-free facilities, with *ad lib* food and water. All animal studies were approved by the UK Home Office under the Animals (Scientific Procedures) Act 1986. Animals were allocated to one of four treatment or control groups and exposed to 45 min renal ischemia, or sham operation, ±MitoQ. Kidneys from healthy animals not subjected to anesthesia or surgery were taken as a baseline control. Under isofluorane general anesthesia, animals underwent laparotomy and exposure of the renal hilum bilaterally. MitoQ (4 mg/kg; added as MitoQ adsorbed to β-cyclodextran (MS010)) in 100 µL 0.9% saline was injected into the tail vein 15 min before the onset of ischemia. The dose was based on reported safety and efficacy data [22]. DecylTPP, which is similar to MitoQ but without the ubiquinone moiety (Fig. 1), was used as a control. Vascular clips (8 mm, interFocus Fine Science Tools, Cambridge, UK) were placed over both renal hila to induce bilateral renal ischemia. At the end of ischemia the clips were removed and kidney reperfusion noted as return of blush color and visualization of flow from the renal vein under microscopy.

Animals underwent either ischemia only (0 min reperfusion) or ischemia followed by reperfusion for 60 min under anesthesia, or for 24 h; the 24 h reperfusion groups were recovered following reperfusion and the abdomen closed with 5.0 Monocryl suture (Ethicon, United States). At the end of reperfusion, all animals were euthanized by cervical dislocation under anesthesia. A blood sample was collected using a heparinized syringe, centrifuged (4000*g* for 10 min at 4 °C) and plasma was analyzed for creatinine using an automated biochemical analyzer (Siemens Dimension RxL Analyser, Siemens AG, Healthcare Division, Germany) at the Core Biochemical Analysis Laboratory, Addenbrooke's Hospital, Cambridge. Kidney tissue was flash frozen in liquid nitrogen and stored at − 80 °C for later analysis.

### Measurement of mitochondrial oxidative damage

To assess damage to mtDNA, total DNA was isolated from frozen kidney tissue (~20 mg wet weight) using the Qiagen DNeasy Tissue Kit (Qiagen, UK), quantified using the PicoGreen dsDNA Assay Kit (Invitrogen) diluted to 3 ng/µL in TE buffer (10 mM Tris, 1 mM EDTA, pH 7.5 (HCl)) and stored at 4 °C. Damage to mtDNA was then assessed using a quantitative PCR method [35,36]. In this method damage to DNA blocks progression of the polymerase during PCR, reducing amplification for a long target (~10 kb) sequence relative to a short target (~200 bp) that is used to control for mtDNA copy number. Each PCR reaction was in a total volume of 50 µL, comprising 15 ng DNA template, 35 µL PCR Mastermix (100 ng/µL BSA, 200 µM NTPs, 20 pmol forward primer, 20 pmol reverse primer, 0.9 mM Mg(OAc)_2_, 12.55 µL H_2_O) and 1 U r*Tth* DNA polymerase XL (GeneAmp, Applied Biosystems). The primer sequences were: forward primer (long and short) 5′-GCCAGCCTGACCCATAGCCATAAT, reverse (short) primer 5′-GCCGGCTGCGTATTCTACGTTA, reverse (long) primer 5′-GAGAGATTTTATGGGTGTAATGCGG. The PCR parameters for the short mitochondrial target were 18 cycles of 30 s at 94 °C, 45 s at 64 °C, 45 s at 72 °C, 10 min at 72 °C. Parameters for the long mitochondrial target were 1 min at 94 °C followed by 16 cycles of 15 s at 94 °C and 12 min at 64 °C, followed by 10 min at 72 °C [37]. Each sample was amplified in duplicate, along with a 50% dilution and a non-template control. The number of PCR cycles was selected so that the reaction remained within the linear phase, defined as amplification of 5 ng DNA producing 40–60% of that of amplification of 10 ng untreated control [37]. Reactions that meet this criterion were then quantified by the Picogreen assay, corrected for the non-template control. Amplification of the long PCR target was then normalized to that of the short target and compared to an untreated or sham operated control.

Total tissue protein carbonyl concentration was determined using an ELISA method using the BioCell PC test kit (Biocell Corp, Auckland, New Zealand) [38]. Tissue samples were first homogenized [39] and the supernatant collected for protein quantification against a bovine serum albumin (BSA) standard and assayed in triplicate (BCA Protein Assay kit, Pierce) prior to carbonyl determination.

### Statistics

Data are presented as means±SEM. Student's unpaired *t*-test and analysis of variance (ANOVA) tests for difference between groups were performed using GraphPad Prism software (v5.0a). A *p* value of <0.05 was taken to be significant.

## Results

### MitoQ protects against IR induced kidney damage

**Fig. 2:**
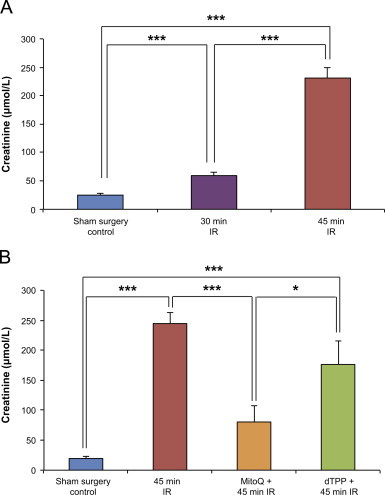
Renal ischemia–reperfusion (IR) injury in a mouse model. (A) 45 min bilateral renal ischemia followed by reperfusion resulted in a significant increase in creatinine at 24 h compared to sham operated controls (laparotomy only) and 30 min ischemia (one-way ANOVA, *p*=<0.0001, with Bonferroni's post-testing between groups where ***p<0.001). (B) Intravenous administration of MitoQ 15 min prior to the onset of 45 min bilateral renal ischemia, significantly protected against kidney injury, as demonstrated by a lower creatinine at 24 h. Administration of decylTPP (dTPP) without the ubiquinone antioxidant moiety did not protect against IR injury. One-way ANOVA; *p*=<0.0001, with Bonferroni's post-testing between groups where *p<0.05, ***p<0.001. *n*=4–5/group. Data are means±SEM.

To investigate whether MitoQ protected against kidney IR damage *in vivo* we used an established mouse model of renal IR injury [1,40]. To assess the impact of IR injury on kidney function we measured plasma creatinine 24 h after IR injury and found that 30 min ischemia led to a mild increase in creatinine while 45 min ischemia led to a far greater increase (Fig. 2A), indicative of significant kidney injury. We therefore used 45 min ischemia to evaluate the efficacy of MitoQ in reducing kidney injury. For this a bolus of MitoQ was given 15 min before the onset of ischemia by a single tail vein injection (Fig. 2B). MitoQ significantly reduced creatinine measured 24 h after reperfusion, while the control compound dTPP did not (Fig. 2B). Therefore IR injury damages kidney function and MitoQ significantly decreases this impairment.

### MitoQ acts by decreasing mitochondrial oxidative damage

**Fig. 3:**
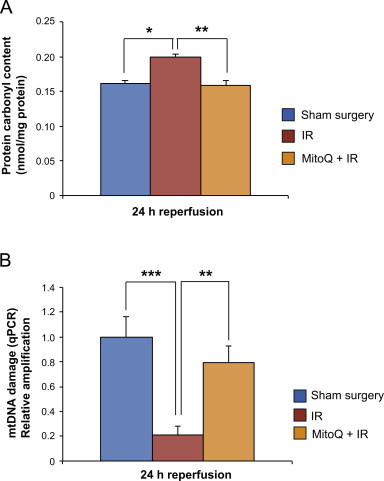
Oxidative damage to proteins and mtDNA following renal ischemia and 24 h reperfusion. (A) Protein carbonyl content in the kidney compared between sham operated groups, IR only and MitoQ+IR groups (one-way ANOVA; *p*=0.0046). Bonferroni's post-testing demonstrated a significant difference between the sham surgery and IR groups at the level of *p*<0.05, and between the IR and MitoQ+IR groups at the level of *p*<0.01. (B) mtDNA damage was measured using a quantitative PCR technique. The sham-operated control was normalized to one and relative amplification of mtDNA in the IR and MitoQ groups compared to this. There was a significant difference in mtDNA amplification between groups 24 h post IR injury (one-way ANOVA; *p*=<0.0001), suggesting significant damage to mtDNA had occurred. Bonferroni's post-testing demonstrated a significant difference between the sham surgery and IR groups at the level of *p*<0.001, and between the IR and MitoQ+IR groups at the level of *p*<0.01. *n*=4/group. *p<0.05, **p<0.01, ***p<0.001. Data are means±SEM.

We next assessed whether the protection by MitoQ was due to decreasing oxidative damage. Protein carbonyl levels, a widely-used marker of oxidative damage, were increased in kidney homogenates after 45 min ischemia and 24 h reperfusion, compared to sham operated controls (Fig. 3A). Treatment with MitoQ prior to ischemia decreased the protein carbonyl content to the level of controls (Fig. 3A). To focus on changes in mitochondria damage we next assessed damage to mtDNA by a qPCR assay [35]. The basis of the assay is that amplification of a long segment (~10 kbp) of mtDNA will be far more disrupted by random damage than a short segment (~200 bp). Consequently the relative amplification of the long and short mtDNA segments indicates the extent of mitochondrial oxidative damage [35]. A major advantage of this assay is that there is no requirement to purify mitochondria from the tissue as oxidative damage and MitoQ treatment are likely to affect the yield and quality of the isolated mitochondria, potentially skewing the interpretation of assays based on isolated organelles. This assay showed extensive mitochondrial damage 24 h after IR injury, and indicated that MitoQ decreased this damage significantly (Fig. 3B). These findings are consistent with a major increase in mitochondrial oxidative damage within the kidney upon IR injury that underlies organ dysfunction and which is prevented by MitoQ.

### Timing of mitochondrial damage and protection by MitoQ

**Fig. 4:**
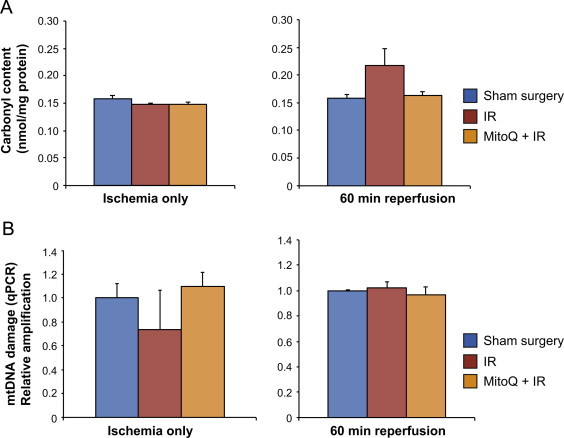
Oxidative damage to the kidney at the end of ischemia and after 60 min reperfusion. (A) At the end of 45 min ischemia (left graph) there was no difference in protein carbonyl content between the sham group and the 45 min IR group or the MitoQ treated group (one-way ANOVA). After 60 min reperfusion (right graph) there was no significant difference between groups (one-way ANOVA). (b) mtDNA damage was measured using a quantitative PCR technique. As before, the sham operated control was normalized to one and relative amplification of mtDNA in the IR and MitoQ groups compared to this. There was no significant difference in mtDNA damage levels between the groups at the end of ischemia or after 60 min reperfusion (one-way ANOVA). *n*=4/group. Data are means±SEM.

To investigate whether mitochondrial damage during kidney injury occurred during ischemia, reperfusion or both, we assessed how much damage occurred during ischemia alone (Fig. 4A and B). Ischemia alone did not affect protein carbonyl levels or mtDNA damage (Fig. 4A and B). Interestingly, there was little damage to mtDNA when measured after 60 min reperfusion (Fig. 4B), compared to 24 h reperfusion, implying that mtDNA damage occurred over a long period following initial reperfusion. These data suggest that mitochondrial damage continues during the reperfusion process.

## Discussion

The role of mitochondria in the initiation and propagation of renal IR injury has attracted increasing interest in recent years. It has become clear that mitochondria are critical early responders to the anoxia and reoxygenation that characterize IR injury, initiating a broad range of responses which affect bioenergetic status, calcium handling, induction of cell death pathways, autophagy and activation of the inflammasome. Importantly from a therapeutic perspective, many of these processes are triggered or augmented by mitochondrial oxidative damage during reperfusion, when mitochondria are both sources and targets of cellular Reactive Oxygen Species (ROS). In this study, we have demonstrated that administration of a mitochondria targeted antioxidant MitoQ, reduces the severity of IR injury to the kidney by decreasing oxidative damage.

The diagnosis of clinical AKI due to IR is typically based on an elevation in plasma creatinine. There is a lag period between the onset of kidney injury with the subsequent rise in creatinine typically peaking ~24 h after the ischemic insult [41]. Creatinine levels at 24 h were therefore measured in our study as a marker of functional kidney injury. We found that 45 min of bilateral renal pedicle ischemia resulted in a significant rise in serum creatinine at 24 h, consistent with the functional injury observed in moderately severe AKI. This allowed us to evaluate MitoQ in a clinically relevant injury model. Using this model, we demonstrated that administration of MitoQ prior to the onset of ischemia provided functional protection to the kidney, presumably through its effect in reducing oxidative damage to the renal tissue at reperfusion. As MitoQ has already been safely used in Phase II clinical trials [27,28], its role as a potential therapeutic agent in clinical IR injury to the kidney warrants further exploration.

## Conflicts of interest

MPM holds patents in the area of mitochondria-targeted antioxidants and is a consultant for Antipodean Pharmaceuticals Inc., which is developing MitoQ as a potential pharmaceutical.
